# Lower GDNF Serum Level Is a Possible Risk Factor for Constipation in Patients With Parkinson Disease: A Case–Control Study

**DOI:** 10.3389/fneur.2021.777591

**Published:** 2022-01-13

**Authors:** Gang Chen, Yinzhen Du, Xue Li, Piniel Alphayo Kambey, Li Wang, Ying Xia, Chuanxi Tang, Mingyu Shi, Li Zai-li, Xin Zai-e, Qin Xiao-ling, Gao Dian-shuai

**Affiliations:** ^1^Department of Neurobiology, Xuzhou Key Laboratory of Neurobiology, Xuzhou Medical University, Xuzhou, China; ^2^Department of Neurology, The Affiliated Shuyang Hospital of Traditional Chinese Medicine of Yangzhou Medical University, Suqian, China; ^3^Department of Neurology, The Affiliated Hospital of Xuzhou Medical University, Xuzhou, China; ^4^Department of Neurology, Xuzhou Central Hospital, Xuzhou, China; ^5^Department of Geriatrics, Shanghai Fourth People's Hospital, Tongji University School of Medicine, Shanghai, China

**Keywords:** GDNF (glial cell line-derived neurotrophic factor), Parkinson's disease, constipation, gastrointestinal dysfunction, enteric nervous system (ENS)

## Abstract

**Background:** Constipation is a significant symptom of Parkinson's disease (PD). Glial-derived neurotrophic factor (GDNF) is important for the morphogenesis of the enteric nervous system and plays a critical role in the preservation of mucosal integrity under enteric glia surveillance. The aim of this work was to evaluate the serum levels of GDNF in patients with PD with and without constipation.

**Methods:** This work included 128 patients with PD. The patients were classified into three groups: those with PD but no constipation (nCons-PD) (*n* = 49), those with prodromal stage constipation (Cons-Pro-PD) (*n* = 48), and those with clinical stage constipation (Cons-Clinic-PD) (*n* = 31). The association between serum GDNF concentration and constipation was explored using logical regression.

**Results:** The nCons-PD group's mean GDNF levels were 528.44 pg/ml, which was higher than the Cons-Pro-PD group's 360.72 pg/ml and the Cons-Clinic-PD group's 331.36 pg/ml. The results of binary logistic regression indicated that GDNF was a protective factor in the prevention of constipation. Cons-Clinic-PD group had a higher score of MDS-UPDRS-II, MDS-UPDRS-III, MDS-UPDRS-IV, and a higher H-Y staging as compared with nCons-PD group. Relative to the nCons-PD group, Cons-Clinic-PD had higher NMSS scores, lower MoCA and PDSS scores, and were more likely to have RBD.

**Conclusions:** GDNF serum levels are lower in patients with PD who are constipated. A low GDNF level is a potential risk factor for constipation in patients with PD.

## Introduction

Parkinson's disease (PD) is the second most prevalent neurodegenerative condition, affecting 2–3% of the population over the age of 65 (1) worldwide and 1.7% of the population over the age of 65 years in China (2), and it is an incurable multisystem condition that causes severe morbidity and healthcare burden. PD is currently diagnosed based on the existence of motor defects such as bradykinesia, rigidity, and tremor, which normally manifest unilaterally or asymmetrically (3). The motor features are primarily the result of dopaminergic neuron failure in the substantia nigra pars compacta (SNc), and by the time of clinical diagnosis of Parkinson's disease, patients have usually already undergone significant neuronal loss (4).

Although the clinical effects of dopamine deficiency are used to diagnose PD, this disease is associated with other neurotransmitter deficiencies that are known to trigger a variety of motor and non-motor symptoms (NMS) and indications. The symptoms and signs, such as hyposmia, REM sleep behavior disorder (RBD), depression, and gastrointestinal (GI) dysfunction, have gained increasing attention and have an effect on the quality of life (QoL) (5). GI dysfunctions are the most common NMS of PD, affecting ~65% (6) of the patients and having a significant negative effect on the QoL. Constipation, bloating, drooling, dysphagia, nausea, vomiting, and gastroparesis are common GI symptoms that can occur up to 20 years before motor symptoms (7). Constipation is a significant symptom of PD and one of the first (NMS) to appear during the prodromal phase (8). Constipation in PD is caused by slowed passage of fecal material through the colon, which is seen in up to 80% of the patients (9). It can occur up to 15 years before motor symptoms and is included in the research criteria for prodromal PD diagnostics as one of the risk factors for potential PD development (10). It has been reported that intestinal smooth muscle cells and enterocytes secrete GDNF (11, 12). GDNF is a member of the transforming growth factor-superfamily that can promote and defend the survival of several different types of neurons, including dopaminergic neurons, motor neurons, sensory neurons, and intestinal neurons. In the intestine, glial cells are an important component of intestinal cells. They are found underneath epithelial cells in the gastrointestinal mucosa, they influence epithelial barrier function, and GDNF was previously believed to be primarily secreted by enteric glial cells. The correlation between constipation in patients with PD and glial cell line derived neurotrophic factor is elusive. In patients with mild cognitive impairment and Alzheimer's disease, decreased peripheral serum GDNF levels have been observed (13). For the past few decades, our laboratory has focused on investigating the protective effects of GDNF on dopaminergic neurons. According to one of our studies, low serum GDNF levels predict cognitive impairment in PD (14). As such, multiple studies have explored the association between PD and intestinal nervous system, intestinal glial cells, and constipation, but it is perplexing whether GDNF serum level is a risk factor in patients with PD with constipation.Here, we report an association of GDNF serum level and constipation in patients with PD using logistic regression model. Results predict that GDNF is a protective factor in the prevention of constipation implying that a low GDNF level is a risk factor for constipation in'patients with PD.

## Methods

### Study Setting and Subjects

Between October 2018 and August 2020, subjects were recruited from the clinic or as in-patients at the Department of Neurology, Xuzhou Central Hospital/Clinical Hospital of Xuzhou Medical University, China. Two experienced neurologists gathered and analyzed extensive demographic details, medical history, disease course, motor symptoms, and non-motor symptoms.

Inclusion criteria for patients with PD were (1) Age ≥18 years old, (2) ability to complete all neuropsychological, clinical, and behavioral tests under the supervision of a physician, as well as to listen, talk, read, and comprehend, (3) two qualified neurologists independently diagnosed PD using the UK Brain Bank Criteria (15), with reference to the Movement Disorder Society diagnostic criteria (16). Secondary parkinsonism caused by drugs, head trauma, vascular disease, or another cause, Parkinsonism and other neurodegenerative disorders, patients with organic digestive tract disorders and a history of digestive tract surgery, systemic diseases such as heart, liver, and renal disease, as well as other diseases that may impair GI function, were excluded.

### Ethics Approval

The Ethics Committee of the Xuzhou Central Hospital in China authorized this study (approval No. XZXY-LJ-20190307-008). The subjects themselves signed informed consent documents.

### Sample Collection

Patients were asked to fast beginning from 22:00 for samples to be collected the next morning. Five milliliters of blood was collected from each patient (between 07:00 and 08:00). The samples were centrifuged for 10 min at 4°C at 1,000 g. The samples were placed at room temperature for up to 2 h before centrifugation. To avoid destroying the serum components, they were immediately dispensed into 130 uL Eppendorf tubes and processed at −80°C for later assays. After collecting serum from all subjects, GDNF levels in patients with PD were determined using enzyme-linked immunosorbent assay kits in strict compliance with the manufacturers' instructions.

### Data Collection

Movement disorder specialists conducted in-person interviews to gather demographic, general information, and clinical data. Two among them clinically evaluated PD subjects in an “ON” state. Standard methods for measuring daily levodopa equivalent doses were used to compare medications (LEDmg). The severity of PD was determined using the Hoehn-Yahr (H&Y) stage and the Movement Disorder Society—Unified Parkinson's Disease Rating Scale. Using the Movement Disorder Society Unified Parkinson's Disease Rating Scale, the study described the tremor dominant (TD) and postural instability/gait difficulty (PIGD) phenotypes of PD (MDS-UPDRS). The PD non-motor symptoms scale (PD-NMSS), Hamilton anxiety scale (HAMA), Epworth sleepiness scale (ESS), rapid eye movement sleep behavior disorder screening questionnaire (RBD-SQ), restless leg syndrome (RLS) diagnosis, Parkinson's disease sleep scale (PDSS), minimental state examination (MMSE), and Montreal cognitive assessment were used to assess non-motor symptoms (MoCA). The ROME IV functional constipation criteria were used to describe constipation. In this study, patients were divided into three groups: those who have PD with constipation (Cons-PD) and those who do not have constipation (nCons-PD). We also asked if constipation occurred before or after the onset of motor symptoms in the Cons-PD population. Patients with constipation before the onset of motor symptoms were referred to as the prodromal stage constipation group (Cons-Pro-PD), and those with constipation after the onset of motor symptoms were referred to as the clinical stage constipation group (Cons-clinic-PD). The Patient Assessment of Constipation–Quality of Life Questionnaire (PAC-QOL) was used in patients with PD with constipation to assess their physical status, psychological status, worry, social relations, and satisfaction, whereas the Patient Assessment of Constipation-Symptoms (PAC-SYM) questionnaire was used to assess the severity of constipation symptoms mentioned by patients.

### Statistical Analysis

The Kolmogorov–Smirnov test was used to confirm the normal distribution of all data. The normally distributed indices, such as age, age of onset of motor symptoms, body mass index (BMI), and Moca global score, were expressed as the mean standard deviation (SD). Since the data such as education, disease length, HAMA, ESS, PDSS, MMSE, PD-NMSS, HAMA, and HAMD were not normally distributed, they were interpreted as the median (interquartile range) [M (QR)]. When comparing multiple classes, the parameters were analyzed using one-way ANOVA if the data matched the normal distribution. If the data did not correspond to the normal distribution, the Kruskal–Wallis test was used to evaluate the non-parametric comparison between the three classes, followed by the Bonferroni test for pairwise comparison to correct the *P* value and monitor the total likelihood of type I error. To compare variations between categorical variables, Chi-squared tests were used. Constipation risk factors were investigated using binary logistic regression. The significance level was set at *p* < 0.05. SPSS, version 22, was used to analyze the data (SPSS Inc, Chicago, Illinois, USA).

## Results

### Demographic Characteristics

**Figure 1:**
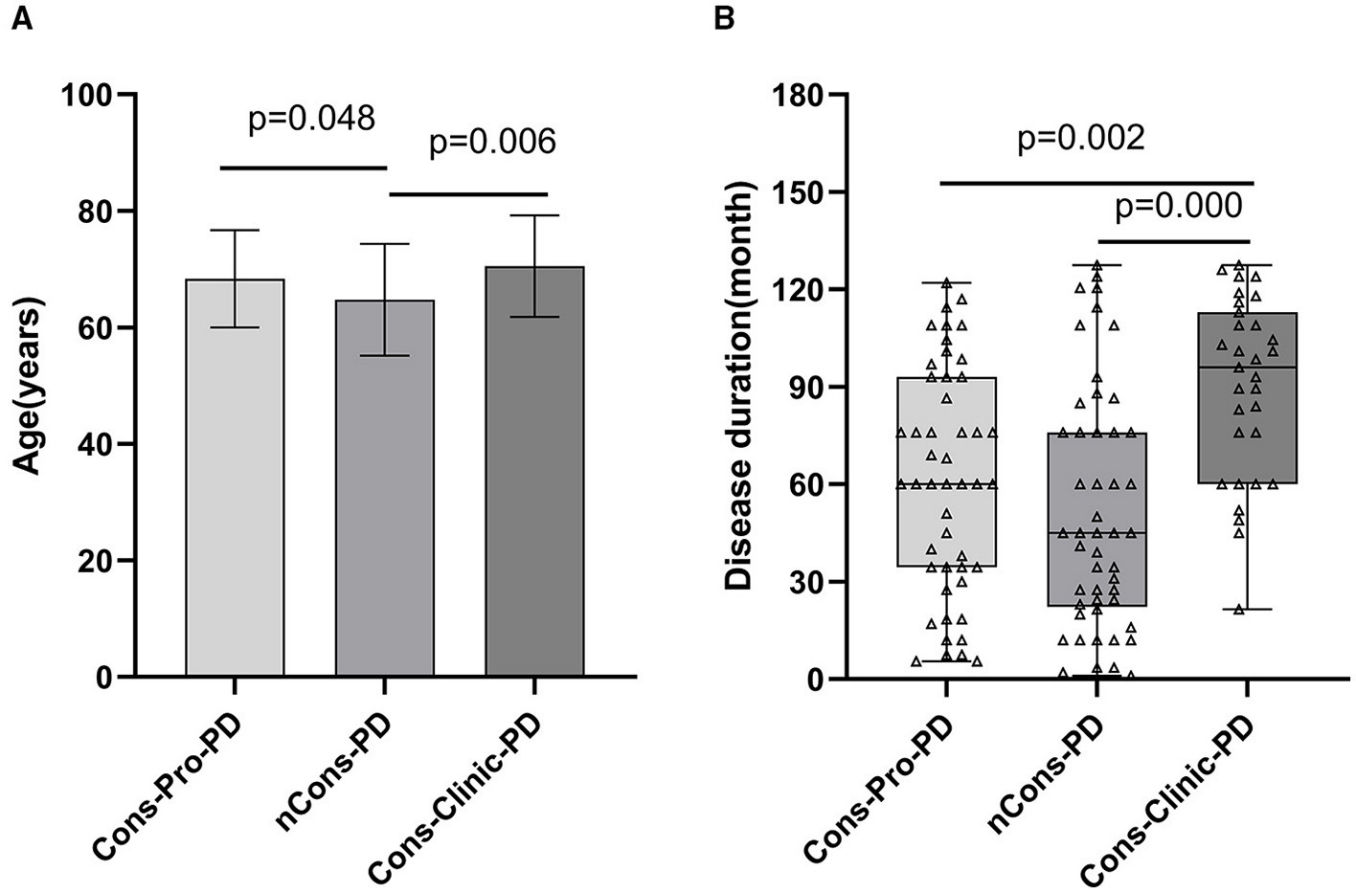
**(A)** compares the ages of the three groups pairwise. The mean age of the nCons-PD group was 64.73 years (SD 9.565, Range 43–82), which was less than that of the Cons-Pro-PD group 68.35 years (SD 8.353, range 49–82, *p* = 0.048) and Cons-Clinic-PD groups 70.52 years (SD8.733, range 51–87, *p* = 0.006), with no statistically significant difference between Cons-Pro-PD and Cons-Clinic-PD groups (*p* > 0.05). **(B)** shows that the median course of disease in the Cons-Clinic-PD group (M 88 months, QR 48–125) was longer than that of the Cons-Pro-PD group (M 48 months, QR 24–84, *p* = 0.002) and the nCons-PD group (36 months, QR 15.5–60, *p* = 0.000), but there was no significant difference in the course of disease between the Cons-pro-PD and nCons-PD groups (*p* > 0.05).

This study included 128 patients with PD who were classified into three groups: those who did not have constipation (nCons-PD) (*n* = 49), those who had prodromal constipation (Cons-Pro-PD) (*n* = 48), and those who did have clinical constipation (Cons-Clinic-PD) (*n* = 31) (Table 1). The study gathered education (years), body mass index (BMI), history of smoking, alcohol use, exposure to insecticides/herbicides, hypertension, diabetes, stroke, brain trauma, family history of Parkinson's disease, and age of motor symptoms onset from all subjects, with no statistically significant differences observed between the groups (*p* > 0.05). Age analysis revealed that the nCons-PD group had a lower mean age (64.73 years) than the Cons-pro-PD group (68.35 years, *p* = 0.048) and the Cons-Clinic-PD group (70.52 years, *p* = 0.006) (Figure 1A). There were no statistically significant variations observed between the Cons-Pro-PD and Cons-clinic-PD groups (*p* > 0.05). Cons-clinic-PD had a longer disease span than the Cons-pro-PD and nCons-PD group (Figure 1B). There were no statistically significant discrepancies between the Cons-Pro-PD and nCons-PD group. It is presumed that the prevalence of constipation in PD is linked to disease progression and age.

**Table 1:** Demographic characteristics of the subjects in the study.

**Group**	**Cons-Pro-PD**	**nCons-PD**	**Cons-clinic-PD**	**Test statistic**	***p* value**
number of patients (*n* =)	48	49	31	–	*p*
Age, (years) [SD, Range]	68.35 ± 8.353 (49–82)	64.73 ± 9.565 (43–82)	70.52 ± 8.733 (51–87)	F = 4.341 (2^#^)*^A^*	**0.015**
Gender (%)					
Female	19 (39.6%)	24 (49.0%)	13 (41.9%)	χ^2^ = 0.925 (2)*^B^*	0.63
Male	29 (60.4%)	25 (51.0%)	18 (58.1%)		
Education (years)	8 (5–8)	8 (5–8.5)	8 (0–8)	χ^2^ = 0.915 (2)*^C^*	0.633
Body mass index (BMI), [SD]	24.15 ± 3.39	24.01 ± 2.88	24.31 ± 3.08	F = 0.088 (2)*^A^*	0.915
Smoking history, (%)	15 (31.3%)	6 (12.2%)	6 (19.4%)	χ^2^ = 5.336 (2)*^B^*	0.069
Alcohol consumption, (%)	10 (20.8%)	5 (10.2%)	4 (12.9%)	χ^2^ = 2.289 (2)*^B^*	0.318
Occupational exposure to insecticidal/herbicides	4 (8.3%)	2 (4.1%)	0 (0.00%)	χ^2^ = 2.993 (2)*^B^*	0.224
History of hypertension, (%)	11 (22.9%)	10 (20.4%)	6 (19.4%)	χ^2^ = 0.166 (2)*^B^*	0.920
History of diabetes, (%)	4 (8.3%)	8 (16.3%)	2 (6.5%)	χ^2^ = 0.202 (2)*^B^*	0.904
History of stroke, (%)	11 (22.9%)	8 (16.3%)	6 (19.4%)	χ^2^ = 0.671 (2)*^B^*	0.715
brain trauma (%)	1 (2.1%)	0 (0.0%)	2 (6.5%)	χ^2^ = 3.476 (2)*^B^*	0.176
PD family history	2 (4.2%)	2 (4.1%)	1 (3.2%)	χ^2^ = 0.051 (2)*^B^*	0.975
Age of motor symptoms onset (years)[SD, Range]	63.66 ± 8.88 (46.5–81.17)	60.18 ± 10.40 (40–81.83)	61.53 ± 10.39 (36–83)	F = 1.538 (2)*^B^*	0.219
Disease duration (month)	48 (24–84)	36 (15.5–60)	88 (48–125)	χ^*2*^ = 20.858 (2)*^C^*	**0.000**

*One-way ANOVA was used to analyze data in ^A^.*

*The LSD exact probability method was used to compare pairs of groups ^C^*.

*Chi-square test on a row multiplied list ^B^. The P value of the pairwise comparison between groups had to be corrected in order to use the Kruskal-Wallis test, and the method was P/3 = 0.0167. p <0.05 indicates that the three groups are not exactly the same, so the next step of pair comparison between the groups is required to determine the reasons for the differences.*

*The ^#^ symbol denotes degrees of freedom. Bold values were statistically significant*.

### GDNF Between the Three Groups

On dopaminergic neurons, motor neurons, sensory neurons, intestinal neurons, and other neurons, GDNF promotes longevity and protects against injury. The peripheral serum GDNF levels of the subjects were measured using the enzyme-linked immunosorbent assay (ELISA) method in this study, and it was discovered that serum GDNF levels in the three groups were not essentially the same (*p* = 0.000). The analysis shows that the Cons-PD group had higher mean GDNF levels (528.44 pg/ml) than the Cons-pro-PD group (360.72 pg/ml *p* = 0.000) and the Cons-Clinic-PD group (331.36 pg/ml *p* = 0.000), with no statistically relevant discrepancies found between the Cons-Pro-PD and Cons-clinic-PD groups (*p* > 0.05) (**Figure 5B**).

### Clinical Characteristics of the Cohort

#### Motor Symptoms and Constipation

**Figure 2:**
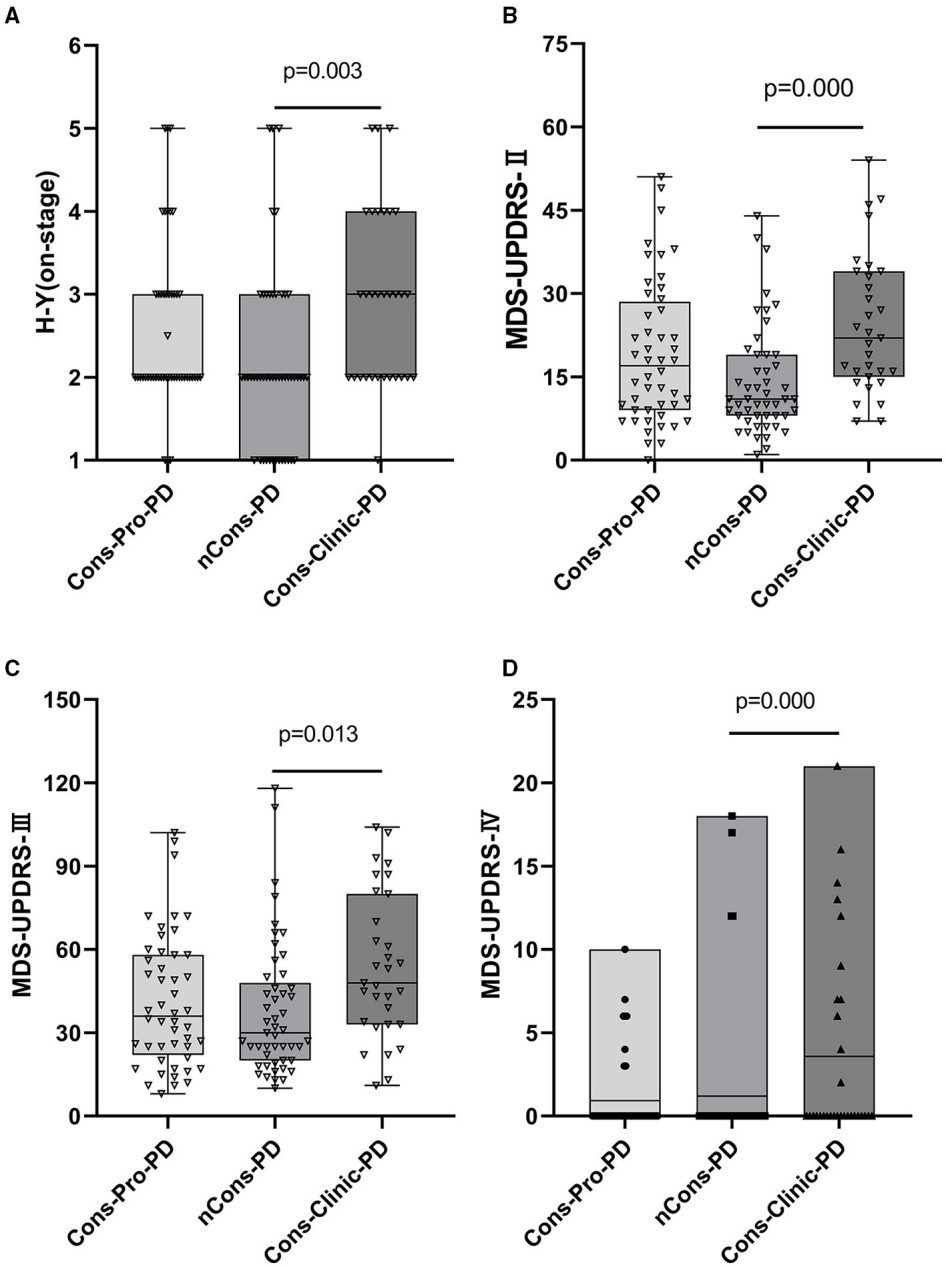
**(A)** Shows that H-Y staging in the three groups is compared. Cons-Clinic-PD had a higher median H-Y staging (M 3, QR 2–4) than the nCons-PD group (M 2, QR 1–3, *p* = 0.003), with no substantial difference in H-Y staging between Cons-pro PD (M 2, QR 2–3) and the nCons-PD group (*p* > 0.05). MDS-UPDRS-II, MDS-UPDRS-III, and MDS-UPDRS-IV total scores were not equal among the three groups (*p* < 0.05). The median MDS-UPDRS-II (M 22, QR 15–34), MDS-UPDRS-III (M 48, QR33–80), and mean MDS-UPDRS-IV (M 3.58, range 0–21) of the Cons-Clinic-PD category were all higher compared with MDS-UPDRS-II (M 11, QR 8–19 *p* = 0.000) **(B)** MDS-UPDRS-III (M 30, QR 20–48, *p* = 0.013) **(C)** and MDS-UPDRS-IV (M 1.204, range 0–18) **(D)**. There was no substantial difference between the Cons-pro-PD group and the nCons-PD group (*p* > 0.05) or the Cons-Clinic-PD group (*p* > 0.05).

In this analysis, there was no statistical difference in the composition ratio among the motor forms of PD (*p* > 0.05) in the general clinical characteristics of the subjects of the three groups (Table 2). MDS-UPDRS-II, MDS-UPDRS-III, and MDS-UPDRS-IV were used to assess motor symptoms and motor complications in patients with PD, and the overall scores were not consistent among the three classes (*p* < 0.05). Further comparison revealed that the differences were primarily reflected in the MDS-UPDRS-II, MDS-UPDRS-III, and MDS-UPDRS-IV scores of the Cons-Clinic-group, which were higher than those of the nCons-PD group MDS-UPDRS-II (*p* = 0.000) (Figure 2B), MDS-UPDRS-III (*P* = 0.013) (Figure 2C), and MDS-UPDRS-IV (*P* = 0.007) (Figure 2D). The median H-Y staging of Cons-Clinic-PD at 3 was higher than the median H-Y staging of nCons-PD at 2 (*p* = 0.003) in H-Y staging for determining PD intensity based on MDS-UPDRS-III motor function score (*p* = 0.003) (Figure 2A). There was no statistical difference in H-Y staging between the Cons-pro-PD and nCons-PD groups (*p* > 0.05) or the Cons-Clinic-PD group (*p* > 0.05). This suggests that in patients with PD, constipation symptoms signify more serious conditions, more noticeable motor symptoms, and more essential motor complications.

**Table 2:** Clinical characteristics of the subjects in the study.

**Group**	**Cons-Pro-PD**	**nCons-PD**	**Cons-clinic-PD**	**Test statistic**	***p* Value**
number of patients (*n* = )	48	49	31	–	*p*
Phenotype				c^2^ = 5.697 (2^#^)*^*B*^*	0.223
PIDG	30 (62.5%)	28 (57.1%)	23 (74.2%)		
Indeterminate	8 (16.7%)	4 (8.2%)	2 (6.5%)		
TD	10 (20.8%)	17 (34.7%)	6 (19.4%)		
H-Y (on-stage)	2 (2–3)	2 (1–3)	3 (2–4)	c^2^ = 11.28 (2)*^*C*^*	**0.004**
MDS-UPDRS-I	11 (7–19.75)	7 (4.5–10.5)	14 (9–18)	c^2^ = 16.439 (2)*^*C*^*	**0.000**
MDS-UPDRS-II	17 (9–28.5)	11 (8–19)	22 (15–34)	c^2^ = 15.36 (2)*^*C*^*	**0.000**
MDS-UPDRS-III	36 (22–58)	30 (20–48)	48 (33–80)	c^2^ = 8.307 (2)*^*C*^*	**0.016**
MDS-UPDRS-IV	0.938 (0–10)	1.204 (0–18)	3.581 (0–21)	c^2^ = 9.580 (2)*^*C*^*	**0.001**
MDS-UPDRS global score	67.5 (40.5–97.5)	51 (33.5–74)	79 (65–126)	c^2^ = 15.349 (2)*^*C*^*	**0.000**
Parkinson's disease therapy					
(LED) mg	443.75 (162.5–600)	337.5 (37.5–400)	537.5 (400–637.5)	c^2^ = 18.491 (2)*^*C*^*	**0.000**
Treatment Naïve	11 (22.9%)	12 (24.5%)	3 (9.7%)	c^2^ = 2.896 (2)*^*B*^*	0.235
Oral levodopa	35 (72.9%)	35 (71.4%)	30 (96.8%)	c^2^ = 8.356 (2)*^*B*^*	0.015
Dopamine agonist	22 (45.8%)	26 (53.1%)	19 (61.3%)	c^2^ = 1.820 (2)*^*B*^*	0.402
Monoamine oxidase B inhibitor	5 (10.4%)	1 (2%)	4 (12.9%)	c^2^ = 3.834 (2)*^*B*^*	0.147
Catechol-O-methyl transferase inhibitor	0 (0.00%)	1 (2%)	1 (3.2%)	c^2^ = 1.392 (2)*^*B*^*	0.498
Anticholinergicagent drugs	2 (4.2%)	6 (12.2%)	3 (9.7%)	c^2^ = 2.076 (2)*^*B*^*	0.354
Amantadine	15 (31.3%)	11 (22.4%)	14 (45.2%)	c^2^ = 4.599 (2)*^*B*^*	0.102
Group	Cons-Pro-PD	nCons-PD	Cons-clinic-PD	Test statistic	*p* Value
Number of patients (*n* = )	48	49	31	–	*p*
NMS global score	44.5 (23.25–73)	29 (15–55.5)	61 (34–90)	c^2^ = 12.487 (2)*^*C*^*	**0.002**
ESS (scores)	10 (1–14)	3 (1.5–10.5)	9 (4–16)	c^2^ = 6.074 (2)*^*C*^*	**0.048**
RBD, case (%)	15 (31.3%)	5 (10.2%)	12 (38.7%)	c^2^ = 9.829 (2)*^*C*^*	**0.007**
PDSS global score	111 (76–128.5)	117 (92–129)	98 (66–113)	c^2^ = 9.347 (2)*^*C*^*	**0.009**
RLS, case (%)	15 (31.3%)	13 (26.5%)	14 (45.2%)	c^2^ = 3.075 (2)*^*B*^*	0.215
PSQI global score	9 (3–13)	8 (4.5–13)	13 (8–15)	c^2^ = 6.585 (2)*^*C*^*	0.037
HAMA (scores)	6 (2–11.75)	4 (1–10.5)	7 (2–14)	c^2^ = 2.266 (2)*^*C*^*	0.322
HAMD (scores)	7 (3–15.25)	5 (3–11.5)	10 (2–16)	c^2^ = 1.157 (2)*^*C*^*	0.561
Moca global score	17.42 ± 5.78	17.33 ± 6.56	13.10 ± 6.37	F = 5.535 (2)*^*A*^*	**0.005**
MMSE	26 (20.75–28)	26 (24–28)	25 (18–27)	c^2^ = 4.210 (2)*^*C*^*	0.122
PACQOL	27 (17.25–2.25)	–	25 (16–47)	Z = −0.121*^*D*^*	0.904
PACSYM	4 (3–5)	–	4 (3–7)	Z = −1.281*^*D*^*	0.2
GDNF (pg/ml)	360.72 ± 93.18	528.44 ± 136.87	331.36 ± 97.74	F = 38.734 (2)*^*A*^*	**0.000**

*One-way ANOVA was used to analyze results in ^A^. The LSD exact probability approach was used to compare pairs of groups^C^. Chi-square test on a row multiplied list ^B^. The p value of the pairwise comparison between groups had to be corrected in order to use the Kruskal–Wallis test, and the method was p/3 = 0.0167. p <0.05 shows that the three groups are not exactly the same, so the next step of pair comparison between the groups is needed to determine the reasons for the variations. The # symbol denotes degrees of independence. Bold values were statistically significant*.

#### NMS and Constipation

**Figure 3:**
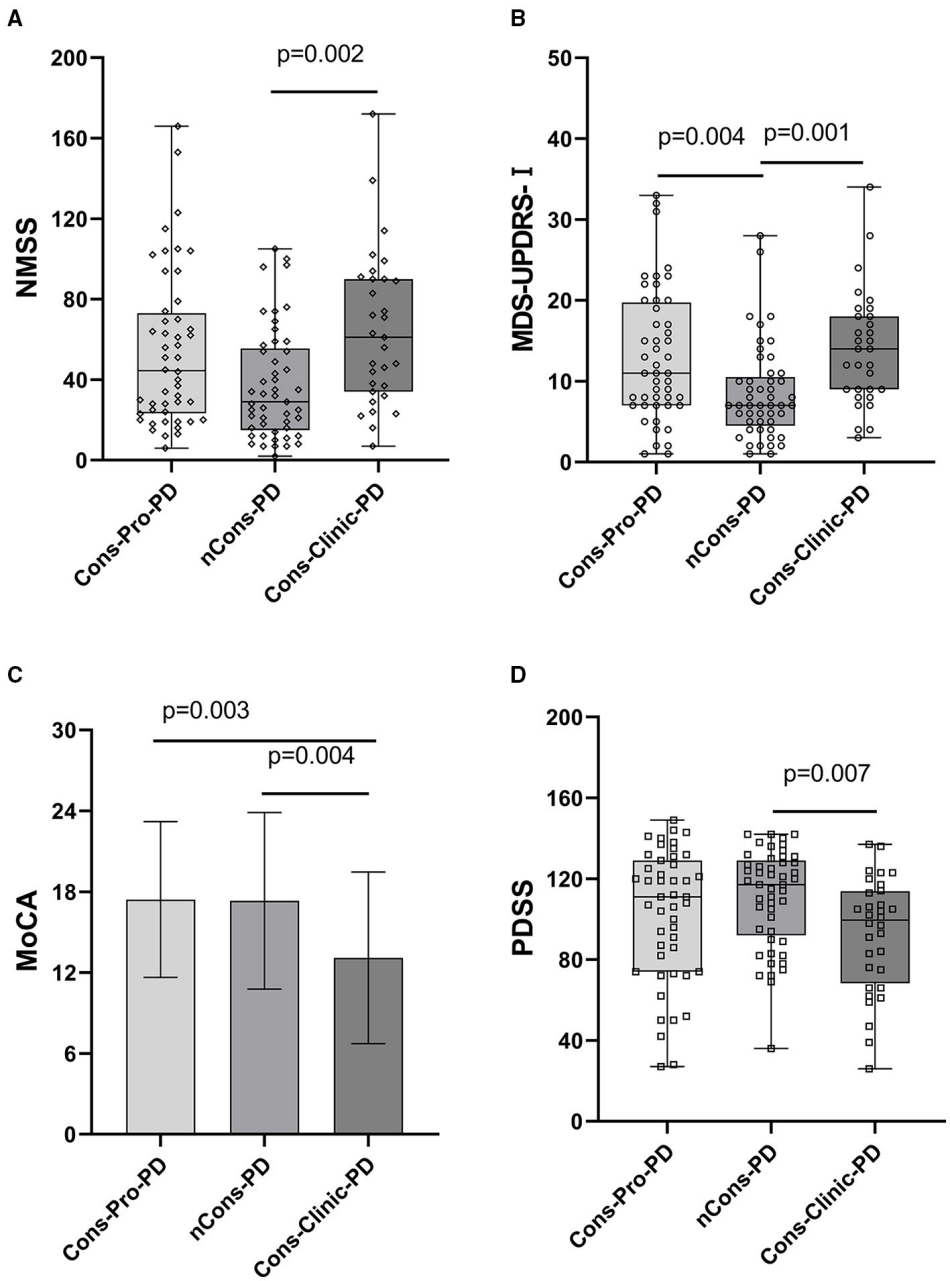
**(A)** Revealed that the median NMSS score of the Cons-Clinic-PD group (M 61,QR 34–90) was higher than that of the nCons-PD group (M 29,QR 15–55.5, *p* = 0.002), although no statistically significant difference was observed in nCons-PD (M 29, QR 15–55.5, *p* > 0.05) and Cons-Clinic-PD (M 61, QR 34–90 *p* > 0.05). **(B)** Revealed that the median MDS-UPDRS-I in the nCons-PD group (M 7,QR 4.5–10.5) was lower than in the Cons-Pro-PD group (M 11, QR 7–19.75, *p* = 0.004) and Cons-Clinic-PD group (*p* = 0.001), and no statistical difference was found between the Cons-pro-PD and Cons-clinic-PD groups (*P* > 0.05). **(C)** Indicates that the mean MoCA of the Cons-Clinic-PD group was 13.10 (SD 6.37) lower than the Cons-Pro-PD group, which was 17.42 (SD 5.78 *p* = 0.003) and 17.33 (SD 6.56, *p* = 0.004). There was no substantial difference in the course of disease between the Cons-pro-PD and nCons-PD groups (*p* > 0.05). **(D)** Revealed that the median PDSS score of the Cons-Clinic-PD group (M 98,QR 66–113) was lower than the nCons-PD group (M 117, QR 92–129, *p* = 0.007), and no statistically significant difference was observed in nCons-PD (M117, QR 92–129 *p* > 0.05) and Cosn-Clinic-PD (M 98, QR 66–113, *p* > 0.05).

Non-motor symptoms such as autonomic nervous symptoms, paresthesia, neuropsychiatric symptoms, sleep disturbances, fatigue, and so on are common in patients with PD. Some non-motor symptoms, such as mental disorders, are closely linked to constipation. This study used NMSS and MDS-UPDRS-I to conduct regular assessments of the subjects' non-motor symptoms to investigate the effect of other non-motor symptoms on constipation in patients with PD. The NMSS and MDS-UPDRS-I total scores were not consistent among the three groups (*p* < 0.05). Further analysis indicated that the variations were primarily expressed in the Cons-Clinic-PD and nCons-PD groups. The NMSS and MDS-UPDRS-I scores in the Cons-Clinic-PD group were higher than those in the nCons-PD group (Figures 3A,B).

While constipation was one of the non-motor symptoms, it appeared that the Cons-Clinic-PD group had more serious non-motor symptoms. Whether the differences in NMSS and MDS-UPDRS-I scores between the three groups are only caused by constipation, and have nothing to do with other non-motor symptoms, we used MMSE and MoCA to evaluate cognition, HAMA, and HAMD to evaluate mental status, and RBD, RLS, and PDSS to evaluate sleep disorders, respectively, and observed the differences between these clinically common non-motor symptoms among the three groups. Simultaneously, the PAC-SYM and PAC-QOL questionnaires were used to measure the magnitude of constipation symptoms and the impact of constipation on everyday life in patients with PD constipation (Table 2).

#### Constipation and Cognition

The MMSE rating did not vary between the three groups of subjects, while the MoCA score indicated that the three groups were not absolutely equal, which may be attributed to the fact that the sensitivity of the MoCA scale to cognitive disability was slightly higher than that of the MMSE.

Further investigation revealed that the mean MoCA of the Cons-Clinic-PD patients was lower than that of the Cons-Pro-PD group (13.10 vs. 17.42, *p* = 0.003) and the nCons-PD group (13.10 vs. 17.33, *p* = 0.004) (Figure 3D). This indicates that cognitive impairment was more severe in the Cons-Clinic-PD group, whereas there was no statistically significant difference in cognitive impairment between the Cons-Pro-PD group and the nCons-PD group (*p* > 0.05).

#### Sleep Disorders and Constipation

**Figure 4:**
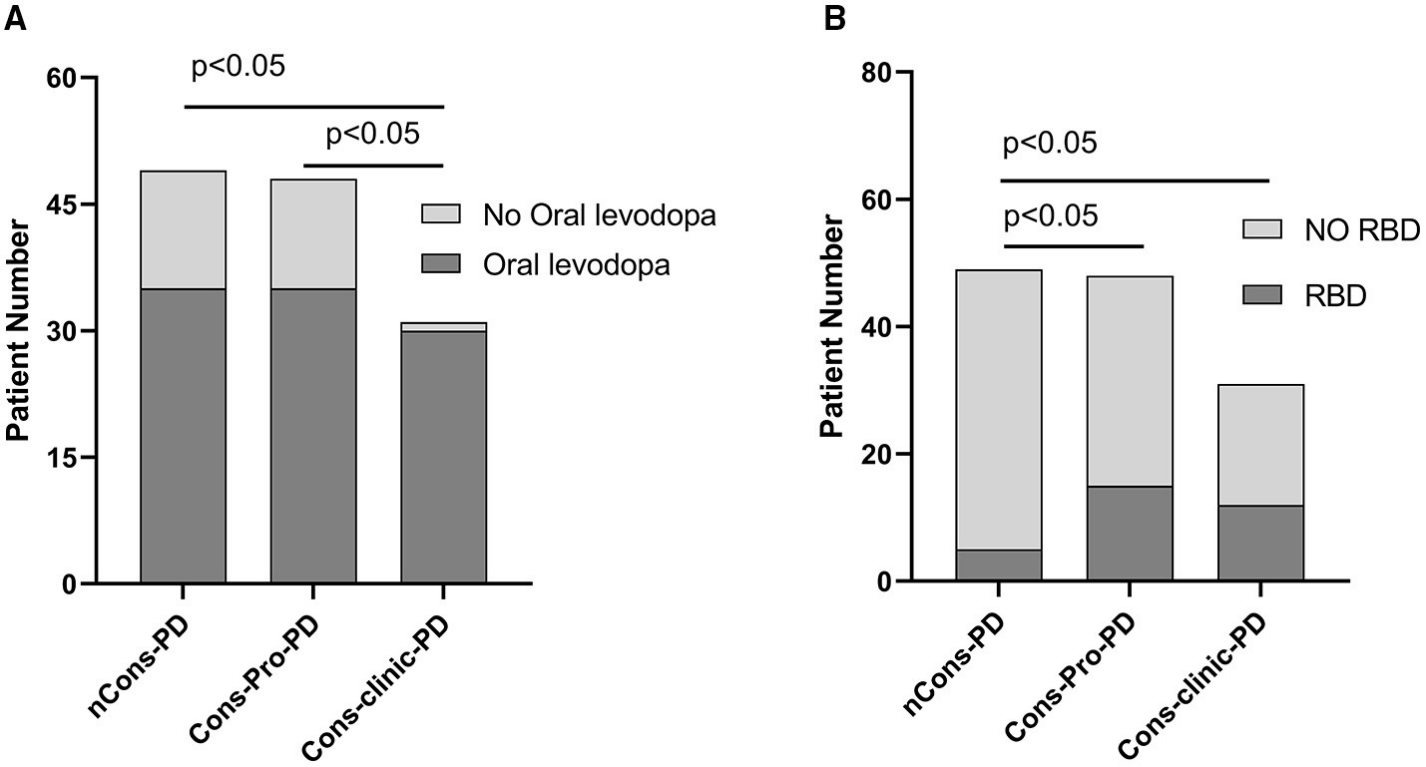
**(A)** Reveals that 96.8% of individuals in the Cons-clinic-PD group use levodopa, which is higher than 72.9% (*p* < 0.05) in the Cons-Pro-PD group and 71.4% (*p* < 0.05) in the nCons-PD group, with no substantial difference (*p* > 0.05) between the Cons-Pro-PD group (72.9%) and the nCons-PD group (71.4%). **(B)** Indicates that 10.2% of people in nCons-PD have RBD, which is lower than 31.3% (*p* < 0.05) in Cons-Pro-PD and 38.7% (*p* < 0.05) in Cons-clinic-PD, but there is no statistical difference between 31.3% in Cons-Pro-PD and 38.7% (*p* > 0.05) in Cons-clinic-PD.

The prevalence of RLS did not vary statistically between the three groups (*p* > 0.05). The ESS was used to assess daytime sleepiness in patients with PD. The three groups were not consistent (*p* = 0.048), but there was no difference between them in a pairwise comparison (*p* > 0.05). The prevalence of RBD differed between the three groups (*p* = 0.007). Further analysis revealed that 10.2% of nCons-PD was lower than 31.3% of Cons-Pro-PD (*P* < 0.05) and 38.7% of Cons-Clinic-PD (*p* < 0.05) (Figure 4B), implying that patients with PD who have constipation are more likely to have RBD. The Cons-Clinic-PD group scored lower on the PDSS than the nCons-PD group (*p* = 0.007) (Figure 3C), indicating that the Cons-Clinic-PD group slept less than the nCons-PD group. There was no statistically significant difference (*p* > 0.05) between Cons-pro-PD and nCons-PD.

#### Constipation, Anxiety, and Depression

Anxiety and depression have been linked to constipation in the general population, but there was no substantial difference in anxiety or depression among the three groups of patients with PD treated with HAMA or HAMD in this work.

### Relationship Between Constipation and Anti-Parkinson's Medications

**Figure 5:**
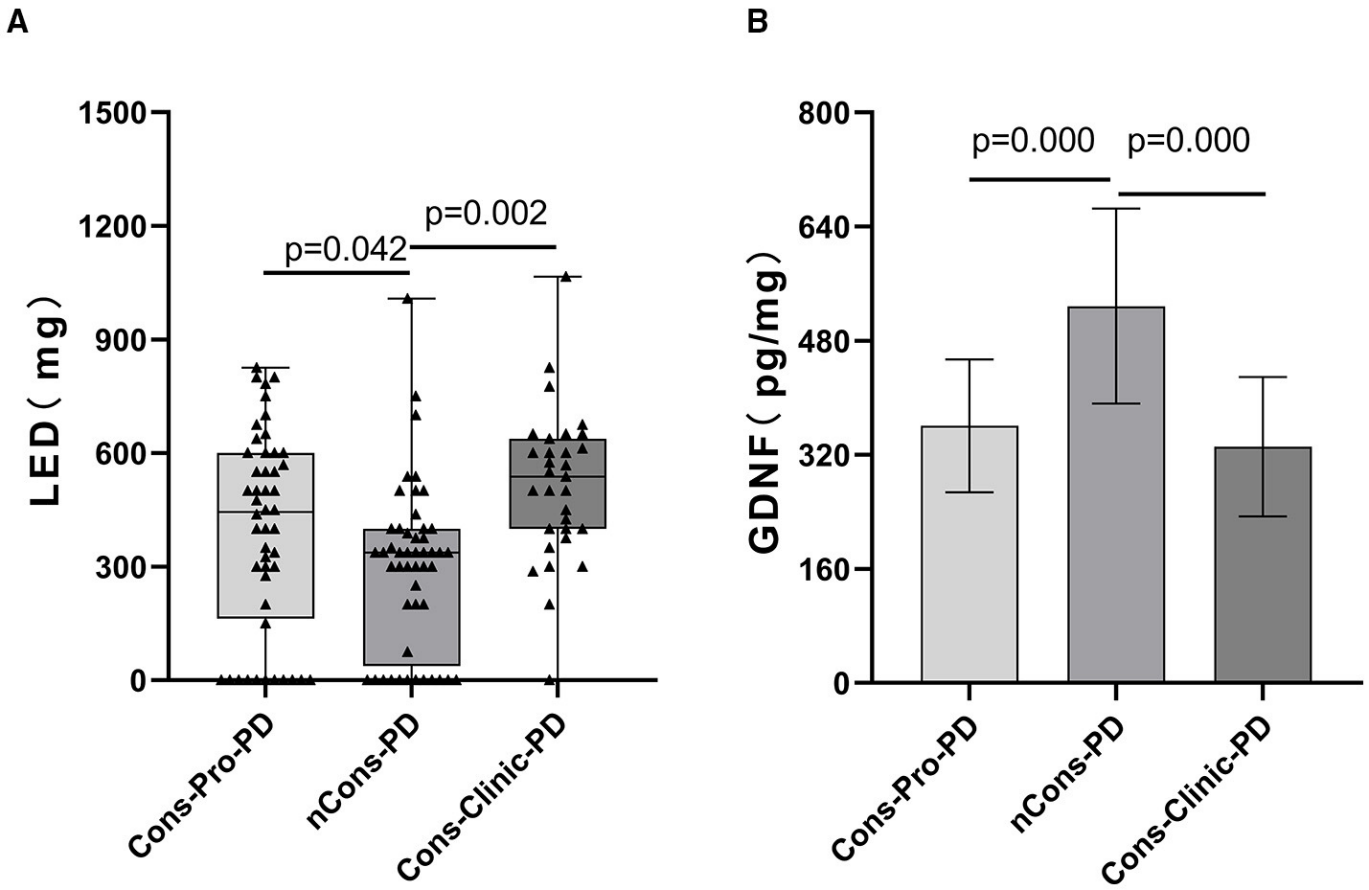
**(A)** Depicts a pairwise analysis of the three groups' median LEDs (mg). The median LEDs (mg) of the nCons-PD group was 337.5 mg (QR 37.5–400), which was lower than the Cons-Pro-PD group (M 443.75 mg, QR 162.5–600, *p* = 0.042) and the Cons-Clinic-PD group (M 537.5 mg, QR 400–637.5, *p* = 0.000), with no statistically relevant difference between the Cons-Pro-PD and Cons-Clinic-PD groups (*p* > 0.05). **(B)** Shows that the mean GDNF levels of the nCons-PD group were 528.44 pg/ml (SD 136.87), higher than the Cons-Pro-PD group 360.72 pg/ml (SD 93.18, *p* = 0.000), and the Cons-Clinic-PD group 331.36 pg/ml (SD 97.737, *p* = 0.000), with no statistically relevant variations found between the Cons-Pro-PD and Cons-Clinic-PD.

Anti-Parkinson's medications can cause constipation, and dopaminergic therapy can also cause constipation. A Cochrane metaanalysis discovered that patients taking dopamine agonists had more constipation than those taking levodopa. There was no substantial difference in the constituent ratios of levodopa, dopa receptor agonists, monoamine oxidase-B-inhibitors (MAO-B), anticholinergic agents, catechol-O-methyltransferase (COMT) inhibitors, amantadine, and not treated with drugs between the three classes in this sample (*p* > 0.05). However, the constituent ratios of levodopa patients and the corresponding dose of levodopa (Levodopa equivalent doses, LEDs mg) are not consistent (*p* = 0.000) (Table 2). LEDs (mg) in the nCons-PD category were lower than in the Cons-Pro-PD group (*p* = 0.042) and the Cons-Clinic-PD group (*p* = 0.002) (Figure 5A). There was no statistically relevant difference between Cons-pro-PD and Cons-clinic-PD (*p* > 0.05). Levodopa was used by 96.8% of the Cons-Clinic-PD group, 72.9% of the Cons-Pro-PD group (*p* < 0.05), and 71.4% of the nCons-PD group (*p* < 0.05) (Figure 4A).

We used the constipation QoL self-rating scale (PAC-QOL) in patients with PD with constipation to assess their physical state, psychological status, worry, social connections, and satisfaction. The Constipation Symptom Assessment Questionnaire (PAC-SYM) was also used to evaluate the seriousness of constipation symptoms recorded by the patients. We compared and analyzed the PAC-SYM and PAC-QOL scores of prodromal and clinical constipation, but no statistical difference was observed between the two classes (*p* > 0.05).

### Constipation Risk Factors

Constipation is correlated with increasing age, female sex, lower socioeconomic status, lower parental education rates, less self-reported physical activity, some drugs, traumatic life events, physical and sexual violence, and depression. We sought to investigate the risk factors for constipation in patients with PD in this study.

A binary logic regression was performed on the subjects with constipation as the dependent variable. The independent variables were sex, age, LED, MDS-UPDRS-I, MDS-UPDRS II, MDS-UPDRS III, MDS-UPDRS IV, H-Ystage, NMSS total score, MoCA total score, RLS, PDSS total score, ESS total score, age of onset of motor symptoms, GDNF, RBD, and the motor forms of Parkinson's disease. The final variables chosen in the model using the forward (wald) approach for data analysis and processing were age, LEDs (mg), MDS-UPDRS-III, H-Y stage, age of motor symptoms, GDNF, and RBD (χ^2^ = 129.945, *p* = 0.000). GDNF is a protective factor in the prevention of constipation after adjusting for age, LED, MDS-UPDRS-III, H-Y stage, age of motor symptoms, and RBD (*B* = −0.106, Wald = 19.486, *p* = 0.000, OR = 0.899, 95% C.I 0.858–0.943) (Table 3). In the discrimination of predicting the impact of constipation, the regression model uses the occurrence of constipation as the endpoint and the prediction likelihood of 0.5 as the discriminant point; the sensitivity for deciding the occurrence of constipation is 96.2%, the precision is 87.8%, and the overall correct rate is 93.0%. Table 3 is the result of binary logic regression with constipation as a dependent variable, suggesting that GDNF is a protective factor in the prevention of constipation.

**Table 3:** Binary logistic regression between PD with constipation and PD with no constipation cohort.

**Variables in the Equation**	**B**	**S.E**.	**Wald**	**df**	**Sig**.	**Exp (B)**	**95% C.I. for EXP (B)**	
							Lower	Upper
Age (years)	−0.45	0.145	9.691	1	**0.002**	**0.638**	0.48	0.846
LED (mg)	−0.006	0.003	5.158	1	**0.023**	**0.994**	0.989	0.999
MDS-UPDRS-III	−0.089	0.035	6.627	1	**0.01**	**0.915**	0.855	0.979
H-Y (on-stage)	−4.573	1.154	15.703	1	**0.000**	**0.01**	0.001	0.099
Age of motor symptoms onset(years)[SD, Range]	0.217	0.102	4.515	1	**0.034**	**1.243**	1.017	1.518
GDNF(pg/ml)	−0.106	0.024	19.486	1	**0.000**	**0.899**	0.858	0.943
RBD	8.025	2.451	10.719	1	**0.001**	**3,056.159**	25.047	37,2901.728

*Bold values were statistically significant*.

## Discussion

Constipation is a frequently occurring NMS of PD, and it is one of the first NMS to appear in the prodromal phase of the disease. The cause of constipation in PD is still uncertain, and the prevalence of constipation varies. The estimated incidence of constipation in patients with PD diverges widely across individual studies, ranging from 8 to 70% in PD (17). The overall incidence of constipation in this work was 61.7%, which was consistent with previous works.

In 2003, Braak and colleagues proposed that PD pathology may begin in the digestive tract and spread to the brain through the vagus nerve (18), and much work has since been done to validate this theory. What is clear, however, is the existence of Lewy bodies (and/or a-synuclein inclusions and Lewy neurites) in PD at almost every stage of the GI tract (19). Enteroendocrine cells in the gut epithelium, which face the lumen and are linked to enteric nerves, have been identified as a possible site where different toxic substances may interact with a-syn, eventually leading to the assembly and spread of pathological a-syn to the myenteric plexus (20). A new mouse model (21) was developed recently to support the Braak hypothesis of the temporal and stereotypical spread of LBs pathology from gut to brain, leading to clinical features of idiopathic Parkinson's disease, including both motor and NMS. Pathological a-syn preformed fibrils were injected into the duodenal and pyloric muscularis layers in that study. Pathologic a-syn spread in the brain was first found in the dorsal motor nucleus, then in caudal parts of the hindbrain, like the locus coeruleus, and much later in the basolateral amygdala, dorsal raphe nucleus, and the SNc. Furthermore, dopaminergic neuron loss, as well as motor and NMS, were observed in a related temporal pattern. This may be a pathological cause of constipation in some patients with PD prior to the onset of motor symptoms. Nonetheless, our basic understanding of the underlying causes of constipation is minimal, and it is debatable to what extent the enteric nervous system itself degenerates in Parkinson's disease.

Glial cell line-derived neurotrophic factor is essential for the regulation of the intestinal barrier. Several studies have found that EGC-derived GDNF improves tight junction organization in intestinal epithelial cells (22). GDNF reduces inflammation-induced impairment of ntestinal epithelial barrier (IEB) function caused by DSG2 loss through p38 MAPK–dependent phosphorylation of cytokeratin (23). As a result, GDNF promotes homeostasis and wound healing in the gut epithelium. Moreover, there are studies embarked on GDNF serum level vs. neurological diseases. For example, Tang and colleagues revealed a low BDNF and GDNF serum level in Chinese male patients with schizophrenia (24). A similar study by Xiao et al. reported a GDNF serum level reduction in patients with cognitive impairment (25). GDNF serum level has enormously implicated in many other neurological disorders (26–28). These are consistent with our findings which revealed a reduced GDNF serum levels in patients with PD who are constipated.

The gut microbiota is increasingly being recognized as a possible player in understanding the pathogenesis and response to treatment in patients with PD (29). A study reported that the gut microbiota is required for the development of motor deficits, reduced intestinal transit, and a-synuclein pathology in a-synuclein-overexpressing mice (30). This research also found that fecal microbiota transplantation from patients with PD exacerbated motor symptoms in germ-free mice, implying that the gut microbiota plays a role in the control of a-synucleinopathy and movement disorders. Short-chain fatty acids and extracellular fibers, such as curli, formed by microbes in the gastrointestinal tract, have been shown in studies to cause a-syn aggregation and motor dysfunction (30). Our findings indicate that serum GDNF levels are lower in patients with PD who are constipated. We propound that low GDNF levels cause more easily impaired intestinal mucosal barrier function, more difficult intestinal mucosal repair, changes in intestinal permeability, and a more susceptible intestinal nervous system to intestinal flora, all of which may be linked to the “second hit” of Braak theory, which is one of the causes of PD constipation. The clinical symptoms of patients with PD with constipation are more apparent after motor symptoms, leading one to speculate that the presence of constipation symptoms aggravates the effect of microflora on the intestinal tract and promotes disease development.

In adults, the prevalence of constipation seems to increase with age and is higher in elderly patients than in younger adults, possibly owing to the degeneration of epithelial, muscle, and neural cells of the colon and pelvic floor (31). This study discovered that the average age of PD without constipation was lower than that of the Cons-Prod-PD group and the Cons-clinic-PD group, implying that age is a risk factor for constipation and that the incidence of PD constipation is also related to age, which is consistent with the previous survey. Meanwhile, we discovered that the duration of disease in the Cons-clinic-PD group was longer than in the Cons-Pro-PD group and nCons-PD group, implying that the risk of constipation increased as the disease course was prolonged. The analysis of gender differences in the occurrence of constipation in PD is not entirely consistent. Some studies have indicated that the incidence of constipation is slightly higher in women with PD, but it has also been stated that the incidence of constipation in men is slightly higher. According to a recent metaanalysis, the prevalence of constipation was not significantly associated with sex (32), and this study did not find that the incidence of constipation is linked to gender.

Slow passage constipation is characterized as decreased motility and frequency of mass movements, resulting in general slow transit throughout the colon, whereas outlet obstruction is defined as a lack of relaxation of the puborectalis muscle or anal sphincters during defecation. Both forms have been studied and verified in'patients with PD (33); however, there is still a lack of direct evidence linking the onset and progression of PD and motor symptoms. Khedr and colleagues investigated moderate-stage PD and discovered that 64% of the akinetic-rigid population was constipated vs. 49% in the tremor group (34). In a review of *de novo* PD, these figures were 45 vs. 21% (35). However, this study found no difference in the occurrence of constipation between activity types.

According to a metaanalysis, the occurrence of constipation is correlated with a rise in clinical disease phases (17). In this study, it was found that the H-Y staging in the clinical stage of PD group with constipation was higher than that in the non-constipation group, and the MDS-UPDRS-II, MDS-UPDRS-III, and MDS-UPDRS-IV scores in the clinical stage of PD group were all higher than those in the non-constipation group. These findings indicate that patients with PD with constipation symptoms have a more serious illness, motor symptoms are more apparent, and motor problems are more severe.

Non-motor symptoms (NMS) are, by all accounts, very common in people with PD. In two trials, nearly 100% of patients reported at least one non-motor symptom (36). Symptoms other than motor function, which Dr. Parkinson describes as NMS, are sleep disturbances, gastrointestinal dysfunction, bladder dysfunction, and even fatigue (extreme exhaustion). PD non-motor symptoms are not independent of one another. Visual hallucinations have been related to perceptual, executive, and sleep dysfunction in Parkinson's disease, and they most likely represent the distribution of Lewy body pathology (37). Anxiety, sometimes in conjunction with depression, may arise prior to the initiation of motor symptoms of Parkinson's disease, implying that this symptom is linked to pathology beyond the nigrostriatal pathway. According to one study, stress is a contributing factor for PD constipation (38). Constipation is more common in patients with PD with RBD sleep disturbance (39). In this work, when NMS were compared between groups, it was discovered that NMS were more severe in the Cons-clinic-PD group, and cognitive and sleep problems were more noticeable in the nCons-PD group than in the nCons-PD group. The incidence of RBD was lower in the nCons-PD party. We hypothesize that constipation develops after the clinical symptoms of Parkinson's disease, and that NMS, such as cognitive dysfunction and sleep disturbances, develop more rapidly.

Constipation is a frequent complication of many PD medications, including anticholinergics, dopamine agonists, and dopaminergic treatments (40). The LED (mg) of the nCons-PD group was lower than that of the Cons-Pro-PD and Cons-clinic-PD groups in this analysis. In the Cons-clinic-PD group, 96.8% used levodopa, compared with 72.9% in the Cons-Pro-PD group and 71.4% in the nCons-PD group, which was consistent with the previous survey.

In conclusion, the motor and NMS were more noticeable in the Cons-clinic-PD group than in the nCons-PD group. We postulate that constipation predicts a faster course of PD in patients. In this work, we selected to examine general motor symptoms, non-motor symptoms, substance use, as well as cognitive, sleep, anxiety, and depressive symptoms. In clinical work, these metrics are common, easy to get, and cost-effective, and they would not imperil patients throughout the assessment process. This study also investigated if serum GDNF might be utilized as a predictor. In the established regression model, our data showed that GDNF had a protective function in PD constipation symptoms, and that low levels of GDNF were a risk factor for PD constipation symptoms.

## Data Availability Statement

The original contributions presented in the study are included in the article/supplementary material, further inquiries can be directed to the corresponding author/s.

## Ethics Statement

The studies involving human participants were reviewed and approved by the Ethics Committee of the Xuzhou Central Hospital in China. The patients/participants provided their written informed consent to participate in this study.

## Author Contributions

GC, QX-l, and GD-s conceived the project and designed the study. GC wrote the manuscript. GC, YD, and YX performed the experiments. PK provided scientific input and English-editing work. GC, LW, LZ-l, XZ-e, and XL performed clinical peripheral blood samples collection and scale evaluation from PD patients. GC, CT, and MS contributed to analysis. All authors contributed to the article and approved the submitted version.

## Funding

This work was supported by the National Natural Science Foundation of China (Grant No: 81971006, to DS).

## Conflict of Interest

The authors declare that the research was conducted in the absence of any commercial or financial relationships that could be construed as a potential conflict of interest.

## Publisher's Note

All claims expressed in this article are solely those of the authors and do not necessarily represent those of their affiliated organizations, or those of the publisher, the editors and the reviewers. Any product that may be evaluated in this article, or claim that may be made by its manufacturer, is not guaranteed or endorsed by the publisher.
